# Retinaldehyde Dehydrogenase Inhibition-Related Adverse Outcome Pathway: Potential Risk of Retinoic Acid Synthesis Inhibition during Embryogenesis

**DOI:** 10.3390/toxins13110739

**Published:** 2021-10-20

**Authors:** Kichul Cho, Sang-Moo Lee, Jina Heo, Yong Min Kwon, Dawoon Chung, Woon-Jong Yu, Seung Seob Bae, Grace Choi, Dae-Sung Lee, Youngjun Kim

**Affiliations:** 1 Department of Genetic Resources Research, National Marine Biodiversity Institute of Korea (MABIK), Seocheon 33662, Korea; kichul.cho@mabik.re.kr (K.C.); jichi9@mabik.re.kr (Y.M.K.); dwchung@mabik.re.kr (D.C.); woonjong_yu@mabik.re.kr (W.-J.Y.); ssbae@mabik.re.kr (S.S.B.); gchoi@mabik.re.kr (G.C.); 2 Department of Applied Bioscience, Dong-A University, Busan 49315, Korea; tkdanwkddy@naver.com; 3 Department of Growth Engine Research, Chungbuk Research Institute (CRI), Chungju 28517, Korea; jaheo514@cri.re.kr; 4 Environmental Safety Group, Korea Institute of Science and Technology (KIST) Europe, Campus E 7.1, 66123 Saarbrücken, Germany

**Keywords:** adverse outcome pathway, retinaldehyde dehydrogenase, retinoic acid, embryogenesis, toxicity

## Abstract

Retinoic acid (RA) is one of the factors crucial for cell growth, differentiation, and embryogenesis; it interacts with the retinoic acid receptor and retinoic acid X receptor to eventually regulate target gene expression in chordates. RA is transformed from retinaldehyde via oxidization by retinaldehyde dehydrogenase (RALDH), which belongs to the family of oxidoreductases. Several chemicals, including disulphiram, diethylaminobenzaldehyde, and SB-210661, can effectively inhibit RALDH activity, potentially causing reproductive and developmental toxicity. The modes of action can be sequentially explained based on the molecular initiating event toward key events, and finally the adverse outcomes. Adverse outcome pathway (AOP) is a conceptual and theoretical framework that describes the sequential chain of casually liked events at different biological levels from molecular events to adverse effects. In the present review, we discussed a recently registered AOP (AOP_297_; inhibition of retinaldehyde dehydrogenase leads to population decline) to explain and support the weight of evidence for RALDH inhibition-related developmental toxicity using the existing knowledge.

## 1. Introduction

Retinoic acid (RA) is an important biological metabolite synthesized from the retinol content (known as “vitamin A”) via a sequential cellular process in the retinoid signaling pathway (RSP) [1]. RSP-mediated RA biosynthesis is a vital physiological process in chordates, since RA interacts with the nuclear receptor superfamily, namely nuclear RA receptors (RARs) and retinoid X receptors (RXRs), bound to the RA response elements (RAREs) in the promoter region of RA target genes [2,3,4,5]. RA effectively regulates gene expression activating the nuclear receptors upon ligand binding, and in the process, eventually influences embryonic development [6,7,8,9]. Therefore, RA plays significant roles in cell growth, differentiation, patterning, and organogenesis during early embryonic development [7,10,11,12]. However, since common animals have no retinol (vitamin A) synthesis mechanism, they obtain retinol in the forms of carotenoid and retinyl esters from their food, and RA is synthesized therefrom through the sequential process of RSP from the retinol content stored mainly in the liver, lungs, kidneys, and bone marrow [1]. The stored retinol is bound to retinol-binding protein 4 (RBP-4) in the serum and enters the cells via interaction with the STRA6 receptor [13]. Subsequently, the transferred retinol-RBP complex is metabolized to retinal through the reaction of retinol dehydrogenases (RODHs) or alcohol dehydrogenases (ADHs) and finally converted to RA by retinaldehyde (or retinal) dehydrogenase (RALDHs). The latter belongs to the oxidoreductase family and plays a critical role in RA synthesis; therefore, this enzyme is considered to be one of the key regulators of RA-related retinol metabolism and embryonic development [14,15]. Unexpected RALDH inhibition, by reducing chemicals, can potentially cause abnormal early embryonic development, and diverse RALDH inhibitors, such as WIN 18446, nitrofen, 4-biphenyl carboxylic acid, bisdiamine, and SB-210661, have been identified till date [16,17]. These chemicals can cause reproductive toxicity in animals; therefore, monitoring and management of RALDH-inhibitors are required in order to resolve the potentially emerging concerns regarding both the aquatic ecosystem as well as human health.

Adverse outcome pathway (AOP) is a conceptual framework describing the biological causal linkages across a molecular initiating event (MIE) due to chemical exposure, key events (KEs) in response to MIE, and adverse outcomes (AO) at individual or population level [18]. A well-organized and integrated knowledge-based AOP framework can potentially be a powerful tool for chemical safety evaluation and alternative animal testing, description of the biological modes of action, and predictive toxicology through quantitative structure-activity relationship (QSAR) modelling [18,19,20,21]. For effective information management of AOP frameworks, the Organization for Economic Co-operation and Development (OECD) had previously launched a new program for the development of AOP using the AOP database known as AOPwiki. AOPwiki platform systematically manages and describes registered AOPs via crowdsourcing [https://aopwiki.org/ (accessed on 14 October 2021)]. In 2020, a new AOP “Inhibition of retinaldehyde dehydrogenase leads to population decline” (AOP ID: 297; AOP_297_) was registered in AOPwiki, and it described the toxicity of RALDH inhibitors due to their potential adverse effect in developmental process [https://aopwiki.org/aops/297 (accessed on 14 October 2021)].

To logically support and explain the weight of evidence of AOP_297_, the present review has integrated all existing knowledge regarding RALDH inhibition-related potential developmental toxicity.

## 2. Toxicological Importance of Adverse Outcome Pathway

**Figure 1:**
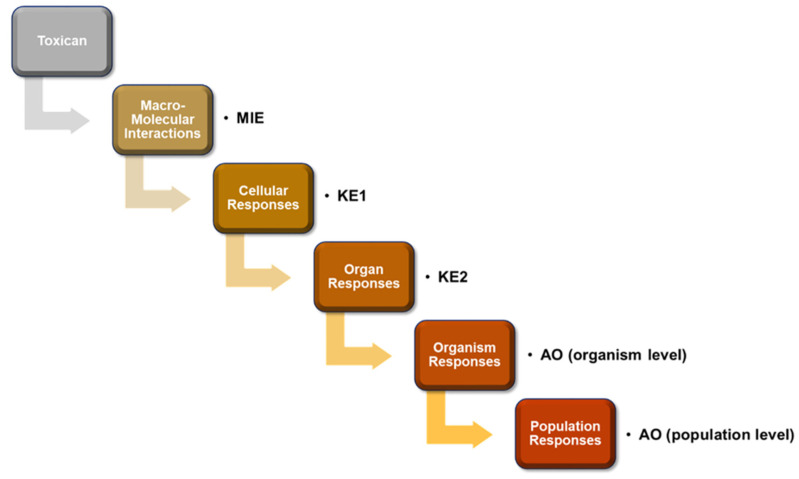
Schematic representation of adverse outcome pathway (AOP) framework. Exposure to toxic chemicals sequentially induce biological toxic events, including molecular initiating events (MIE), key events (KEs), and finally adverse outcomes (AOs) at organism or population level.

In vivo animal toxicity test, using rats, rabbits, fishes, and mice, is considered a useful conventional method to evaluate the potential toxic effects of known or unknown chemicals in humans [21]. However, since the establishment of the “3 Rs”principle, referring to the reduction, refinement, and replacement of animal use, by Russell and Burch [22], animal testing has become limited due to ethical problems and social issues [23,24,25]. Therefore, several alternatives to animal testing methods have been developed since then, including in vitro cell culture, tissue engineering technique, and in silico computer simulation toxicity tests [21,26]. AOP is a conceptual framework explaining the intermediate biological reactions of toxic chemicals from MIE toward KE and AO, and provides a well-organized sequential mode of action for chemical toxicity [27]. As shown in Figure 1, when the toxic agent induces MIE by molecular interactions mediated by receptor binding or enzyme inhibition, subsequent cellular responses (KE1), such as altered gene transcription, relevant protein production, or altered cell signaling occur inducing further organ responses (KE2). The KEs include toxicity at tissue and organ levels, and abnormal development and functions. The sequential KEs ultimately induce AOs at the individual and population levels, including impaired development, population decline, and extinction (Figure 1) [27]. Construction of AOPs requires assessment of the weights of evidence (WoEs) to determine confidence of supporting information via semi-quantitative methods [28].

Since the constructed AOPs are available for the prediction of toxicology of chemicals at molecular, cellular, individual, or population level, it can alternatively replace animal testing models via in silico or in vitro risk assessment [29,30]. Furthermore, AOP can be a promising toxicological tool for fast and accurate chemical risk assessment, since it is useful for the integrated approaches to testing and assessment (IATA) based on mechanistic information [31,32]. OECD had launched a new program for the development of new AOP framework in 2012, and the AOPwiki database has been hosted by the Society for the Advancement of AOP (SAAOP) [https://aopwiki.org/ (accessed on 14 October 2021)]. Till date, almost 400 AOPs have been registered into AOPwiki DB, and SAAOP plays a significant role in promoting the development of AOPs in coordination with OECD Extended Advisory Group for Molecular Screening and Toxicogenomics (EAGMST). OECD also provides a QSAR Toolbox program to assess the hazardous chemicals regarding skin sensitization, based on the information on AOPs [https://www.oecd.org/chemicalsafety/risk-assessment/oecd-qsar-toolbox.htm (accessed on 14 October 2021)]. Consequently, construction of a new AOP framework would provide a piece of key information for the development of in silico risk assessment.

## 3. RA Biosynthesis and Retinoid Metabolism

The all-trans-retinol and its derivatives play a critical role in animal physiology, including reproduction, vision, cell differentiation, and vertebrate development [4,33,34]. The physiological roles of retinol are highly relevant to RA biosynthesis alongside its regulation of gene transcription. RA (all-trans-retinoic acid) is a lipophilic low-weight biomolecule (approximately 300 Da), and a member of the 4000-plus strong family of retinoids, metabolized from the all-trans-retinol (called vitamin A) via sequential reversible and irreversible metabolic processes [1,35].

**Figure 2:**
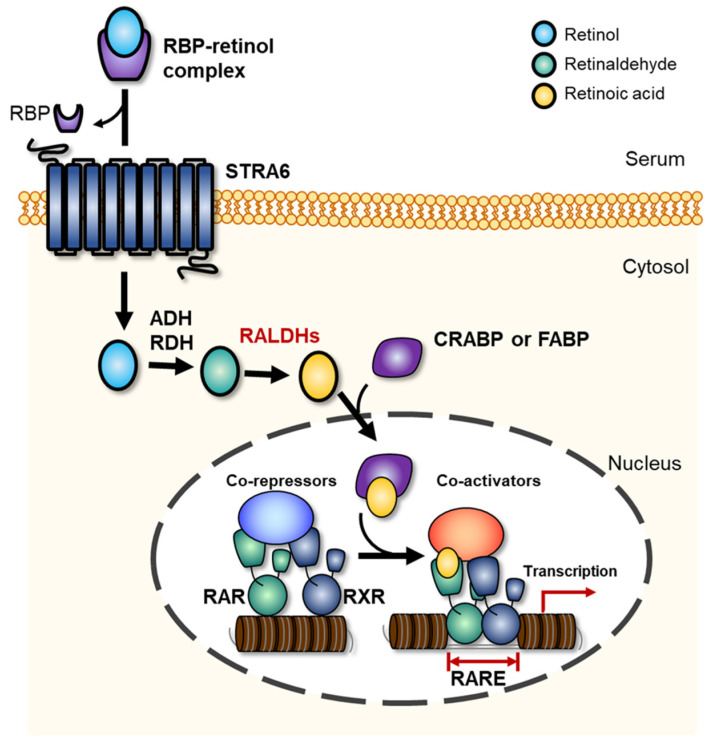
Cellular retinoic acid synthesis and metabolism in chordates. Retinol bound to plasma retinol-binding protein (RBP) is transferred to cytosol via STRA6 receptor, where aldehyde dehydrogenase (ADH) and retinol dehydrogenase (RDH) convert it to retinaldehyde, and finally retinaldehyde dehydrogenases (RALDHs) convert retinaldehyde to retinoic acid (RA). Subsequently, the synthesized RA binds to cytoplasmic retinoic acid binding protein-2 (CRABP-2) and regulates target gene transcription.

A schematic representation of RALDH-mediated paracrine mechanisms of RA biosynthesis pathway from all-trans-retinol is presented in Figure 2. In serum, the all-*trans*-retinol is transiently bound to the secreted 21 kDa retinol-binding protein (RBP), which is a sole specific carrier synthesized within the liver, and RBP-retinol complex enters the cells via the membrane-attached STRA6 receptor, which is a 74 kDa multi-transmembrane domain protein [13,36,37]. Subsequently, the internalized retinol is either stored as retinyl esters or converted to RA [36,38]. Apo-cellular retinol-binding proteins I-III (CRBP I-III) in the cytoplasm transfers retinol to lecithin retinol acyltransferase (LRAT), which is a microsomal enzyme that esterifies CRBP-retinol complex or free retinol to retinyl esters for cellular storage [38,39]. The absorbed retinol is converted to RA in the RA-generating tissue via step-by-step response, as shown in Figure 2 [36]. In the first rate-limiting RA biosynthesis step, plasma retinol converts to retinaldehyde via reversible oxidization by either alcohol dehydrogenase (ADH) or retinol dehydrogenase (RDH) enzyme families [40]. Cytosolic ADH, which belongs to the medium-chain dehydrogenase/reductase family, converts the alcohol form of retinol to retinaldehyde with reduction of nicotinamide adenine dinucleotide (NAD^+^) to NADH [14]. Similarly, RDH, which is a microsomal member of the short-chain dehydrogenase/reductase (SDR) family, also catalyzes the reversible chemical reaction between retinol and retinaldehyde via reduction of NAD^+^ to NADH [14]. In the second step, the produced retinaldehyde is oxidized to RA by irreversible reaction of RALDH, included in the family of oxidoreductase (Figure 2) [36,41,42]. RALDH catalyzes RA synthesis from retinaldehyde according to the following reaction scheme:

Retinal + NAD^+^ + H_2_O ↔RA + NADH + H^+^ (1)

The reaction mechanism between RALDH and retinaldehyde (RAL) theoretically begins with the interaction of RAL’s aldehyde group with a cysteine residue in the enzymatic active site; the reaction forms a thiohemiacetal intermediate, and hydride transfer occurs to form NADH and thioester intermediate (TI) [43,44]. Subsequently, the generated TI reacts with H_2_O, and is more nucleophilic due to a glutamate residue near the active site [45]. This proposed process finally synthesizes RA and NADH from retinal, NAD^+^, and H_2_O_2_. The three types of RA receptors (RAR-α, ß, and γ) and RXRs are switched on via RA binding and act as a transcription factor for target gene expression (Figure 2) [46,47]. The synthesized all-trans RA binds to RAR with a higher binding affinity than to RXR [46]. Allenby et al. [46] had previously determined the binding affinities of 9-cis-RA to RXRs and RAR by saturation kinetics and Scatchard plot, and showed approximately 20-fold higher binding affinity between RA and RAR (0.1–0.7 of K_d_ value) compared to that between RA and RXR (14.1–18.3 of K_d_ value) in the mouse. The synthesized RA binds to cytoplasmic retinoic acid binding protin-2 (CRABP-2) and regulates target gene transcription [48].

The transcriptional regulation induced by the binding of RA to RARs and RXRs is systematically controlled at cellular level. The synthesized or extracellularly derived RA can either be degraded to non-active forms by cytochrome P450 enzyme family or bind to nuclear receptors via the cellular RA-binding protein II (CRABP-II), as shown in Figure 2. In the nucleus, RXR forms homodimers, whereas RAR can form heterodimers upon reacting with RXR. The synthesized dimers can bind to RA response elements (RAREs) located in the RA target gene promoter and modulate target gene expression [5].

The target gene expression process by RAR-RXR heterodimer (RRH) and RA binding have been well-described in a published review [2]. In brief, the unliganded RRH represses its associated gene transcription before RA binding reaction. The RRH recruits co-repressors, including nuclear receptor co-repressor (NCOR) 1 and 2 (also known as thyroid hormone- and retinoic acid receptor-associated co-repressor 1, 2, TRAC-1, 2) [2,49]. The co-repressors recruit histone deacetylase protein complex (HDAC) and polycomb repressive complex 2 (PRC2), resulting in histone methylation, chromatin condensation, and gene silencing [2]. When RA binds to RRH, a conformational change occurs, and repressive co-activators are replaced. Subsequently, the replaced co-activators recruit histone acetylase complexes and trithorax-group proteins (TrxG) to regulate histone methylation, chromatin relaxation, and gene activation [2]. The detailed transcriptional regulation process induced by RA binding to receptors has been well-described in some previous reviews [2,5,50].

## 4. Pleiotropic Roles of RA and RALDH Inhibitors

RALDHs belongs to the oxidoreductase family and plays a critical role in RA synthesis from the retinaldehyde content; therefore, this enzyme is considered to be one of the key regulators of RA-related retinol metabolism and embryonic development [14,15]. The RALDH may be categorized into different classes of aldehyde dehydrogenase (ALDH) superfamily, including class 1 (cytosolic proteins), class 2 (mitochondrial proteins), class 3 (tumor-related proteins, inducible, or found in the endoplasmic reticulum), and microsomal aldehyde dehydrogenase [51,52,53]. The structures of RALDHs had been determined in previous studies [53,54,55]. RA biosynthesis and signaling seem to be limited to chordates, due to the existence of RALDH [36]. The *ALDH*1*A*1, *ALDH*1*A*2, and *ALDH*1*A*3 genes code cytosolic RALDH enzyme (RALDH1, 2, 3) that catalyzes the synthesis of retinoic acid (RA) from retinaldehyde. The RNA and protein expression patterns of RALDH in human tissues can be verified in the transcriptomic and proteomic database of THE HUMAN PROTEIN ATLAS [56]. According to data, differentially expressed RNA and RALDH levels were determined in human tissue cell lines including brain, eye, endocrine tissues, lung, proximal digestive tract, gastrointestinal tract, liver and gallbladder, pancreas, kidney and urinary bladder, female and male tissues, muscle tissues, adipose and soft tissue, skin, bone marrow and lymphoid tissues, and blood. Among these, male tissues including ductus deferens, testis, epididymis, seminal vesicle, prostate, and female tissues such as the vagina, ovary fallopian tube, endometrium, cervix, uterine, placenta, breast showed relatively higher expressions of RNA and protein expression than the other tissues. Furthermore, endocrine tissues, lungs, and pancreas also exhibited relatively higher enzyme expression levels than those of other human tissues [https://www.proteinatlas.org/ENSG00000128918-ALDH1A2/tissue (accessed on 14 October 2021)]. Meanwhile, in the embryonic cells, three types of cytosolic RALDHs (RALDH1, 2, 3)-coding genes differentially expressed during the early embryogenesis [57]. According to a previous study by Niederreither et al. [57], *ALDH*1*A*1 expression was increased in developing lung, and stage-specifically expressed in stomach and intestine epithelial and mesenchymal layers of embryonic cells. On the other hand, *ALDH*1*A*2was expressed in the kidney nephrogenic zone, and *ALDH*1*A*3 was specifically expressed in the intestinal lamina propria.

Due to the physiological importance, RALDH inhibition by diverse chemicals during the early embryogenesis potentially causes serious disease such as fetal alcohol spectrum disorder (FASD), congenital diaphragmatic hernia (CDH), and Moyamoya disease (MMD) [17,58,59]. Kot-Leibovich and Fainsod [58] reported that the alcohol (ethanol) compete with available RALDH activity during embryonic development, thereby inducing abnormally lowered RA level, and finally FASD. Mey et al. [17] also previously reported that RALDH2 inhibitors including nitrofen, 4-biphenyl carboxylic acid, bisdiamine, and SB-210661 induced posterolateral defects in the rat diaphragm. Furthermore, abnormal organ developments such as eye, kidney, and brain also can be induced by RALDH-inhibition, and thus potentially cause population decline of chordate in the ecosystem. Therefore, the regulation of RALDH inhibitors needs to be re-considered for the improvement of public health.

The physiological importance of RA is explained by pleiotropic modulation, including early embryogenesis, cell differentiation, and innate and adaptive immune homeostasis as shown in Table 1. Among the diverse roles of RA, morphogenesis and organogenesis during vertebrate development have been widely recognized over the past few decades [2,60,61,62,63,64,65]. Wilson et al. [66] had reported that retinoic acid-free embryos exhibited abnormal development of neural tube with alteration in the actin filaments and junctional complexes of the cell layer. Wang et al. [67] had previously shown that RALDH2-deficient mouse embryo exhibited abnormal development of posterior foregut derivatives (stomach and duodenum) along with liver growth. The relationship between mammalian craniofacial morphogenesis and RA signaling had also been reported in a previous review [68]. Furthermore, the RA-mediated *Hox* genes and adhesion molecule-induced skin morphogenesis via determination of axial orientation had been reported in an embryonic chicken skin explant culture [69]. RA-mediated epidermal morphogenesis had been reported by Asselineau et al. [69]. They had shown the delipidized serum-exposed abnormal human keratinocytes to be restored by RA addition, whereas higher RA concentration (>10^−7^ M) reduced epidermal maturation and produced parakeratosis [69]. Multiple roles of RA signaling for mammalian eye development had been reported in previous reviews [70,71]. RA signaling was shown to not only be required for interactions between the invaginating lens placode and optic vesicle, but also to promote the development of ventral retina and optic nerve, thereby modulating eye and photoreceptor development [8,70,71]. Dupé and Lumsden [72] had reported that the RA-mediated transforming signal is involved in the posterior hindbrain structure expansion, and that RA signaling is related to rhombomere boundaries, which are transverse developmental units of the embryonic hindbrain. Morphogenesis during embryonic development is regulated by diverse RA target genes. The enhanced key target genes expressed by RA signaling during early organogenesis include *Hoxa*1, *Hoxb*1, *Hoxa*3, *Hoxd*4, *vHnf*1, *Pax*6, *Olig*2, *Cdx*1, *Pdx*1, *Hoxa*5, *Pitx*2, *Ret*, and *Stra*8, whereas the repressed target genes include *Fgf8* and *TGF-ß1* [36]. These genes are highly related to the vertebrate developmental process. Specifically, over-expression of *Hoxa*1, *Hoxb*1, *Hoxa*3, *Hoxd*4, and *vHnf*1 genes is highly relevant to hindbrain anterior-posterior patterning developmental process, whereas that of *Pax6* and *Olig2* induce spinal cord motor neuron differentiation [36,73,74,75,76]. Repressed *Fgf8* and overexpressed *Cdx*1 genes induce early somite formation, and heart anteroposterior patterning during embryonic development [36,77]. While the induction of meiosis is related to the *Stra*8 gene, anterior eye formation and kidney formation are relevant to the overexpression of *Pitx*2 and *Ret* genes, respectively [36,78].

The roles of RA in cell growth and differentiation and associated immune responses have been reported in previous studies [61,63,79,80,81,82]. Påhlman et al. [61] had reported RA and 12-0-tetradecanoyl-phorbol-13-acetate (TPA), and combination of both, to significantly increase neuron-specific enolase activity and noradrenalin concentration in cultured human SH-SY5Y neuroblastoma cells. Mucida et al. [79] had reported RA to be a key modulator of the cytokine transforming growth factor-beta (TGF-beta)-dependent immune responses promoting the differentiation of anti-inflammatory naïve T cells into regulatory T (Treg) cells. Therefore, RA may be considered an important molecule for animal immunity [79]. Breitman et al. [80] had previously shown that RA effectively induces differentiation of HL-60 cells compared to retinol (vitamin A), retinal, and retinyl acetate. Furthermore, Strickland and Mahdavi [83] had reported the induction of multiple phenotypic changes (morphological alteration, enhanced plasminogen activator production, increased collagen-like protein synthesis, and elevated sensitivity to cyclic AMP compounds) by RA in F9 embryonal carcinoma cell lines, which had lost the capacity of differentiation. The studies revealed that RA is a key molecule for morphogenesis and differentiation of multiple types of cells.

RALDH and ALDH are key enzymes for RA biosynthesis through retinoid metabolism, as described in Section 3. Therefore, RALDH or ALDH activity-inhibiting chemicals, such as disulphiram, phenyl carboxylic acid, bisdiamine, acetaldehyde, bisdichloroacetyldiamine WIN 18446, dichloro-all-trans-retinone (DAR), 4-(diethylamino)benzaldehyde (DEAB), disulphiram, 4-amino-4methyl-2pentyne-1-al (AMPAL), citral, gossypol, and SB-210661 would potentially induce reproductive toxicity [16,17,58,84]. Amory et al. [85] had reported that the bisdichloroacetyldiamine WIN 18446, which is considered a potent inhibitor of the testicular retinal dehydrogenase and aldehyde dehydrogenase, remarkably reduced intratesticular retinoic acid, and significantly impaired spermatogenesis and fertility. Mey et al. [17] had characterized diaphragmatic defect-inducing tetratogens in embryonic rat models that imitate congenital diaphragmatic hernia (CDH) in infants, and verified the characterized chemicals, including nitrofen, 4-biphenyl carboxylic acid, bisdiamine, and SB-210661, to inhibit RALDH2 enzyme-inducing posterolateral defects in the rat diaphragm. Shabtai et al. [86] had reported that acetaldehyde produced from the oxidation of ethanol via embryonic ADH effectively inhibited RALDH2, thereby reducing RA signaling and being responsible for the developmental malformations leading to fetal alcohol spectrum disorder. Perz-Edwards et al. [87] had shown the treatment of DEAB to reduce frontonasal region, retina, hindbrain, and otic vesicle during the development of zebrafish embryo, indicating reduced RA levels to induce abnormal development. In addition, Chute et al. [88] had reported that ALDH inhibition by DEAB treatment delayed human hematopoietic stem cell differentiation, along with reduced RAR-mediated signaling.

The results revealed the ALDH- and RALDH-mediated RA signaling to be highly related to several diseases, including Sjögren-Larsson syndrome (SLS), cancer, Alzheimer’s disease, alcohol flushing syndrome, and hyperprolinemia [16]. Therefore, specific and non-specific RALDH inhibitors are commonly used for the study of relevant human diseases.

## 5. RA Signaling-Associated Eye Development and RALDH-Relevant AOP (AOP_297_)

The reciprocal relationship between RA signaling and mammalian eye development has been well-described over the past few decades [70,89,90,91,92,93].

**Figure 3:**
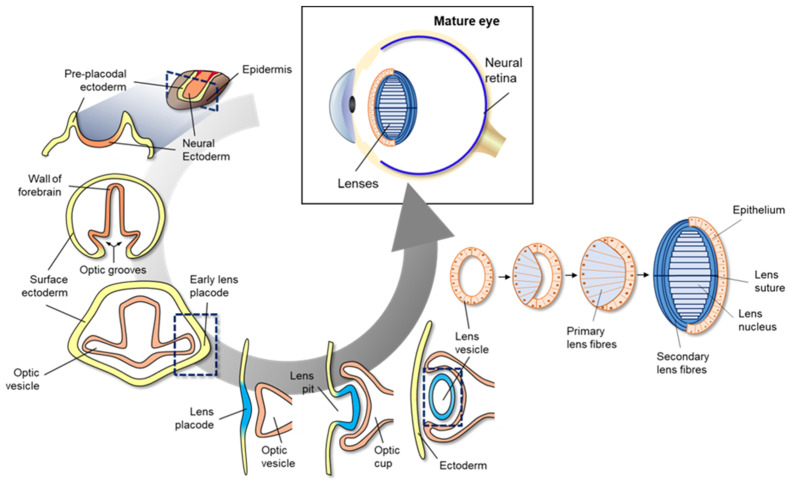
Schematic diagram of mammalian eye development. Eye development in vertebrates occurs in the neural ectoderm origin, surface ectoderm, and neural crest-derived periocular mesenchyme of embryonic cell. Mammalian eye development starts with formation of the wall of the forebrain on either side of the optic grooves. The optic grooves gradually deepen as the neural folds become elevated, and the neural tube is closed by surface ectoderm. The optic grooves extend to the near ectoderm to form early lens placode by thickening of the adjacent area. During the interaction of optic vesicles with the ectoderm, the generated lens placode invaginates to form the lens pit, and optic vesicles invaginate to form the double-layered optic cup. Lens vesicles are generated from the lens pit and are surrounded by the double-layered optic cup; finally, the generated lens vesicle becomes the fetal lens and mature lens after the development of primary lens fibers, secondary lens fibers, lens suture, and lens nucleus.

**Figure 4:**
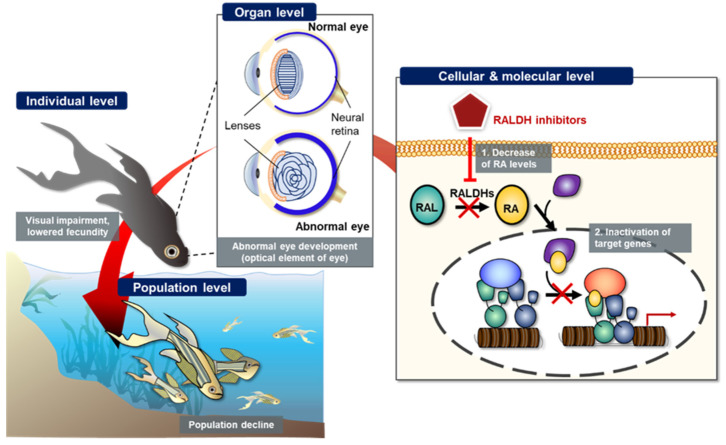
Schematic representation of potential retinoic acid (RA) inhibition-related adverse outcome pathway (AOP). Inhibition of retinaldehyde dehydrogenase (RALDH) by known or unknown inhibitors potentially induces decreased cytosolic retinoic acid (RA) levels, which in turn, changes target gene expression, leads to abnormal embryonic eye development at individual level, and finally results in population decline of teleosts.

Eye development in vertebrates occurs in the neural ectoderm, surface ectoderm, and neural crest-derived periocular mesenchyme of the embryonic cell [70]. As shown in the cross-section of neural ectoderm, pre-placodal ectoderm, and epidermis (Figure 3), mammalian eye development starts with the formation of a wall of the forebrain at either side of the optic grooves. The optic grooves gradually deepen as the neural folds become elevated, and the neural tube is closed by the surface ectoderm. The optic grooves extend to the near ectoderm to form early lens placode by thickening of the adjacent area. During the interaction of optic vesicles with the ectoderm, the generated lens placode invaginates to form the lens pit, and optic vesicles invaginate to form the double-layered optic cup. The lens vesicles are generated from the lens pit, are surrounded by the double-layered optic cup (Figure 4), and finally, become the fetal lens and mature lens after the development of primary lens fibers, secondary lens fibers, lens suture, and lens nucleus. A detailed explanation of vertebrate eye development has been well-described in previous reviews [94,95].

Many previous studies have reported that RA signaling plays a vital role in vertebrate eye development [70,71,89]. RA synthesis is mediated by three types of RALDHs, including RALDH1, RALDH2, and RALDH3. These enzyme-coding genes contribute to RA synthesis in non-overlapping locations during eye development [89]. For instance, when the optic cup forms, the RALDH2-coding gene is first expressed in the perioptic mesenchyme. Subsequently, RALDH1- and RALDH3-coding genes are expressed in the dorsal and ventral retina, respectively [89]. The synthesized RA then participates in eye development to form the optic cup, ventral retina, cornea, and eyelids [89]. Mouse knockout model-mediated genetic studies demonstrated the role of RA in embryonic eye development [89,96]. Raldh1-knockout mice had previously presented no noticeable adverse outcome and only showed slight RA activity in the dorsal retina owing to the compensation of Raldh3 during eye development [89,96]. However, Raldh1 and Raldh3 double knock out mutants induced overgrowth of mesenchyme in the cornea and eyelids due to defective apoptosis [89,97,98]. In another study, the Raldh3 knock out embryo exhibited eye and nasal defects, and thus could not survive due to blockage of the nasal passages [99]. Raldh3 knock out mutant showed abnormal embryonic development by shortening of the ventral retina. Furthermore, the study of Raldh2 knock out mutant and Raldh1/Raldh2 double knock out mutants showed the stoppage of optic cup development, which was subsequently rescued by RA addition [100]. These findings suggested that RALDH is an important molecule for embryonic eye development. A previous study had shown that citral competitively inhibits ventral RALDH of zebrafish embryo, thereby leading to reduced RA synthesis [8]. Citral administration under neurula-stage of the zebrafish embryo led to the deformity of a ventral retina in zebrafish eyes. Conversely, exogenous addition of RA induced a duplication of the zebrafish retina in a concentration-dependent manner during the initial formation of the optic primordia [71,101]. The results together indicated that the RALDH inhibition-associated undermined RA synthesis potentially causes abnormal eye development in chordates, including teleosts.

The potential AO can be sequentially explained from the molecular level to population level via the AOP framework. As shown in Figure 4, we schematized the potential AO by RALDH inhibition. At both cellular and molecular levels, RALDH inhibitors decreased the conversion of retinal to RA, thereby inhibiting the activation of target gene transcription. At the organ level, inhibited RA synthesis led to abnormal eye development, caused by inhibited optical elements. Finally, the abnormal eye development led to visual impairment at the individual level. Previous studies had reported that ocular coloboma, caused by incomplete closure of the choroid fissure, was potentially related to the ventral RA levels due to several genes; for example, *Lrp*6 (a bottleneck coreceptor in the canonical Wnt signaling pathway) knock out mutation resulted in ocular coloboma in mice [89,102]. The Lrp6 null mutant showed a dramatically inhibited level of RALDH in mouse eye [102]. The visual impairment potentially affected reproduction and survival of teleosts, with lowered fecundity and hence decline in the population level (Figure 4). However, the population decline caused by RALDH inhibition-mediated abnormal eye development still remains poorly understood. Therefore, the weak weight of evidence needs to be redeemed to completely construct the AOP framework.

## 6. Conclusions

RALDH is a key enzymatic component in cellular RA synthesis from retinaldehyde. The synthesized RA systematically regulates gene transcription via conversion of RAR or RXR receptors, thereby conducting morphogenesis and other functions during the embryonic development. In the current review, we comprehensively presented the importance of RA-mediated eye development, and potential AO derived from RALDH inhibition alongside the AOP (AOP_297_) framework, based on the existing knowledge.

RALDH-relevant gene deletion and RALDH-inhibitor administration caused abnormal eye development, and could potentially lead to population decline. Despite the weak weight of evidence regarding the relationship between RA inhibition and population decline, the AOP suggested the need for regulation of RALDH inhibitors to prevent potential risk of reproductive toxicity. However, further studies are required to support WoEs to construct a more complete AOP.

## Figures and Tables

**Table 1:** Physiological roles of retinoic acid (RA) in chordate.

	Role of Retinoic Acid (RA)	References
Embryogenesis	Neural tube development	[66]
	Posterior foregut derivatives development and liver growth	[67]
	craniofacial morphogenesis	[68]
	Skin morphogenesis	[69]
	Eye development	[70,71]
	posterior hindbrain structure expansion	[72]
	Early somite formation	[36,77]
	Heart anteroposterior patterning	[36,77]
	Kidney formation	[36,78]
Others (immunity, cell differentiation)	Neuron differentiation	[36,61,73]
	Anti-inflammatory naïve T cells differentiation	[79]
	HL-60 cells differentiation	[80]

## Data Availability

The datasets used and analysed during the current study are available from the corresponding author on reasonable request.
